# Brain-derived neurotrophic factor Val66Met and CYP2B6 polymorphisms as predictors for ketamine effectiveness in patients with treatment-resistant depression

**DOI:** 10.1177/02698811241238284

**Published:** 2024-03-13

**Authors:** Nelson B Rodrigues, David Chen-Li, Joshua D Di Vincenzo, Ashwin Juneja, Benjamin D Pinder, Roger S McIntyre, Joshua D Rosenblat

**Affiliations:** 1 Braxia Health, Mississauga, ON, Canada; 2 Department of Psychology, Neuropsychology Track, University of Windsor, Windsor, ON, Canada; 3 Inagene Diagnostics Inc., Toronto, ON, Canada; 4 Brain and Cognition Discovery Foundation, Canada, Toronto, ON, Canada; 5 Department of Psychiatry, University of Toronto, Toronto, ON, Canada

**Keywords:** Ketamine, treatment-resistant depression, major depressive disorder, pharmacogenetics, tolerability

## Abstract

**Background:**

Converging lines of evidence indicate that ketamine is a rapid antidepressant for individuals with treatment-resistant depression. Hitherto, no reliable a priori predictors of ketamine response have been reported. Pharmacogenetic biomarkers have yielded mixed results regarding potential candidate genes associated with ketamine’s biochemistry as reliable predictors of response.

**Aims:**

No studies have examined the effects of Val66Met and CYP2B6 genotypes on patients receiving repeated infusions of intravenous ketamine.

**Methods:**

In all, 85 participants with major depressive disorder who had previously received four infusions of intravenous ketamine were recruited to the foregoing study. Buccal swabs were collected and genotype variants across the Val66Met and CYP2B6 genes were analyzed. A repeated measures mixed linear model was used to assess change in depressive symptoms, suicidality, and anxiety, correcting for sex and age. Multiple regression was run to determine whether these genetic markers were associated with treatment efficacy for depressive severity, suicidal ideation, anxiolytic response, and degree of dissociation to intravenous ketamine.

**Results:**

Participants experienced significant overall reductions in depression, suicide, and anxiety. Overall, 25% met the response criteria and 15% met the remission criteria. However, Val66Met and CYP2B6 did not significantly predict changes in symptoms of depression, suicide, anxiety, or average dissociation.

**Conclusions:**

This study contributes to the growing literature that ketamine efficacy is unlikely to be predicted by single genes, and a pleiotropic approach may likely be necessary for developing reliable predictors of clinical benefits.

## Introduction

Major depressive disorder (MDD) presents a significant public health and economic concern and is associated with significant disability on an individual level. While there exists a myriad of effective psychopharmacological treatments for MDD, clinicians are forced to rely on “trial and error” approaches to determine the correct treatment type and dose per individual clinical presentation. The current putative treatments for MDD leverage medications that modulate the monoamine system (i.e., serotonin, epinephrine, and dopamine). However, evidence suggests that a significant proportion of patients are unable to achieve remission with these medications (Rush et al., 2006; Trivedi et al., 2006). These patients, classified as treatment resistant, exhibit poor syndromal and functional recovery, a high rate of suicide, and a decreased likelihood of responding to subsequent treatments (Guze and Robins, 1970; Rush et al., 2006).

Notwithstanding the role of monoamines, a growing body of literature has suggested that the glutamatergic system may mediate depressive etiology (Dowlati et al., 2010; McEwen and Stellar, 1993; Slavich and Irwin, 2014). Ketamine, a N-methyl-D-aspartate receptor antagonist, has demonstrated rapid and robust antidepressant and anti-suicidal effects in patients with treatment-resistant depression (TRD) (Coyle and Laws, 2015; McIntyre et al., 2020a; McIntyre et al., 2020b; Singh et al., 2016). The Food and Drug Administration approved an intranasal formulation of (S)-ketamine (i.e., esketamine) under the brand name Spravato, suggesting that ketamine may be a viable treatment option for patients with refractory depression. Nevertheless, real-world effectiveness data suggest that up to 50% of patients with TRD receiving intravenous ketamine will exhibit no clinical improvement in depressive symptoms, and between 1% and 5% will exhibit a worsening of their symptoms (Di Vincenzo et al., 2022; McIntyre et al., 2020a, 2020b). Hitherto, there exist no reliable clinical predictors of ketamine’s antidepressant response and continue to rely on trial-and-error paradigms. As a result, there has been growing interest in elucidating clinical biomarkers that can accurately predict each patient’s response to treatment. To this end, pharmacogenetics (PGx) has emerged as a promising candidate in the field of psychopharmacology.

Numerous preclinical and clinical trials have investigated candidate genes that characterize the metabolism of ketamine. Ketamine is metabolized extensively by the liver by the cytochrome P450 (CYP450) enzymes. The CYP2B6 enzymes account for a large proportion of ketamine metabolization, with minor contributions from CYP3A4 and CYP2C9 (Hijazi and Boulieu, 2002; Yanagihara et al., 2001). Therefore, some groups have hypothesized that loss-of-function in the CYP2B6 alleles may lead to poor outcomes and adverse events (McIntyre et al., 2020a, 2020b; Rong et al., 2018). However, a study of 67 patients with TRD by Zarate and colleagues suggested that there was no association between CYP genotype and antidepressant response (Zarate et al., 2012).

Ketamine is also considered to be a potent mediator of the brain-derived neurotrophic factor-Tropomyosin receptor kinase B-mTOR pathway (BDNF-TrkB-mTOR). Murine studies leveraging a knock-in of the Val66Met BDNF allele suggested that ketamine’s antidepressant response is mediated through this BDNF pathway (Fukumoto et al., 2019). Indeed, BDNF is a principal factor for synaptogenesis and plasticity within the hippocampus and is hypothesized to be a factor in ketamine’s mechanism of action (Autry et al., 2011). It was therefore hypothesized that individuals with the Met BDNF polymorphism, which affects BDNF folding effectively impairing the binding of the neurotrophin to the TrkB receptor, would yield lower antidepressive responses. Human studies, however, have produced mixed results. Laje et al. provided evidence that MDD patients with the Val/Val BDNF allele expression had a stronger antidepressive response compared to Met carriers (Laje et al., 2012). A separate study noted that Val carriers experienced reductions in suicidal ideation at 0.5 mg/kg and 0.2 mg/kg, whereas Met carriers only endorsed an improvement in suicidal thoughts at the 0.5 mg/kg dosage (Chen et al., 2019). Comparatively, a double-blind, randomized, placebo-controlled clinical trial of a single infusion of ketamine did not report any significant differences between Val or Met carriers (Su et al., 2017). Furthermore, it has been well established that the relationship between glutamatergic signaling and BDNF is mediated by glucocorticoid expression through the hypothalamic–pituitary–adrenal (HPA) axis (Dutton et al., 2022). Altered HPA axis activity due to stress has been shown to reduce synaptogenesis and neuroplasticity in several cerebral structures, especially the hippocampus, which, in turn, affects glutamatergic functioning (Duman et al., 2019; Lüscher and Malenka, 2012). A recent systematic review provided evidence that increased HPA axis activity was associated with an increased suicide risk, independent of other psychiatric conditions (Berardelli et al., 2020). Ketamine’s effect on depression and suicidal ideation may be mediated through neuroendocrine response (Wang et al., 2019).

A recent systematic review examined extant literature to identify key allelic variants that impact ketamine metabolism (Meshkat et al., 2021). The review identified 18 genes that had previously been associated with intravenous ketamine. Importantly, the Val66Met BDNF pathway and the CYP450 metabolizer were the most reported genes that affected ketamine metabolism and its clinical response (Meshkat et al., 2021). However, all studies used a single infusion paradigm, which is incongruent with current clinical practices (McIntyre et al., 2021a). No studies, to date, have examined the effects of Val66Met and CYP2B6 genotypes on patients receiving repeated infusions of intravenous ketamine. Herein, we aim to investigate the extent these genes mediate the antidepressant, anti-suicidal, and antianxiety effects of ketamine.

## Methods

### Participants and study design

Participants with treatment-resistant MDD who previously received intravenous ketamine at the Canadian Rapid Treatment Center of Excellence (CRTCE) in Mississauga, Canada were recruited to participate in this study. Eligibility criteria and treatment protocol of patients receiving ketamine have been previously described (McIntyre et al., 2020b). Briefly, patients with treatment-resistant depression (i.e., failure of two or more adequate trials of different antidepressant classes) received four infusions of intravenous ketamine. The initial two infusions were dosed at 0.5 mg/kg, whereas the final two infusions were flexibly dosed between 0.5 and 0.75 mg/kg. Dose optimization to 0.75 mg/kg was dependent on inadequate clinical response and tolerability to the index dose. Prior to each infusion, participants completed a battery of self-report measures that included the Quick Inventory for Depressive Symptomatology Self-Report 16-Item (QIDS-SR_16_) and Generalized Anxiety Disorder 7-Item (GAD-7), which assessed depressive and anxiety severity, respectively. In addition, approximately 15-min post-infusion, nursing staff administered the clinician-administered dissociative states scale (CADSS) to assess dissociative symptoms during infusion.

Following the completion of the four infusions, patients were enrolled in the study presented herein which took place from January 2021 to July 2021. Due to the COVID-19 pandemic, all study procedures took place remotely. To be enrolled in the study, participants must have had a previous diagnosis of treatment-resistant major depression, be between the ages of 18 and 65, have received at least four infusions of ketamine at the CRTCE, have no history of substance use disorder, alcohol use disorder, or psychosis. Once consented to the study, patients were mailed a genetics kit containing saliva swabs from Inagene Diagnostics Inc. which were completed at their homes or during their fourth ketamine infusion appointment.

The Personalized Insights (™) Pain and Mental Health test from Inagene Diagnostics, Inc. (Toronto, Ontario) was used for pharmacogenetic testing on all patients. Genomic DNA was isolated from buccal swabs and the genotypes of 116 variants across 56 genes were assessed using the Agena MassArray platform (Agena Bioscience, San Diego, CA, USA), a robust and highly sensitive tool for SNP and CNV genotyping that uses matrix-assisted laser desorption/ionization-time of flight mass spectrometry technology for resolving oligonucleotides (Ellis and Ong, 2017). Diplotype information and pharmacogenetic associations were assigned by Inagene’s custom algorithms that employ consortia-curated knowledge bases including, but not limited to, the Clinical Pharmacogenetics Implementation Consortium (https://CPICPGx.org) and PharmGKB (https://www.pharmgkb.org/). Metabolizer status for the CYP450 2B6 was delineated into categories, based on their activity. Phenotype classifications included poor metabolizers (PM), intermediate metabolizers, normal metabolizers, rapid metabolizers, and ultrarapid metabolizers (Desta et al., 2019). The classification was based on the number of alleles with no functioning, low functioning, normal functioning, or increased functioning for drug metabolism. For example, the PM classification was based on two decreased or non-functioning alleles including the *6/*6, *18/*18, and *6/*18 diplotypes. The foregoing study was approved by a community institutional research ethics board and registered under the identifier NCT04695405.

### Statistical analysis

An effectiveness analysis was conducted on an intention-to-treat group, wherein all participants received at least one ketamine infusion and had at least one QIDS-SR16, QIDS-SR16 SI, and GAD7 score. Two genes (i.e., Val66Met and CYP2B6) were selected a priori based on a systematic review by our group identifying genetic markers of interest associated with ketamine response (Meshkat et al., 2021). The Hardy–Weinberg equilibrium was assessed using a χ^2^ test for both genes. To assess change in depressive symptoms, suicidality, and anxiolytic symptoms, a repeated measures linear mixed model was used. An unstructured matrix was used and data were estimated using a restricted maximum likelihood approach. The model corrected for sex and age and the alpha was set to 0.05. Multiple comparisons were corrected using a Bonferroni method for post hoc analyses with baseline as the reference category. Responders were calculated as a reduction of QIDS-SR16 Total Score ⩾ 50% from baseline to follow-up and remitters were calculated as a QIDS-SR16 Total Score ⩽ 5 at follow-up.

Multiple regression was run to determine whether these genetic markers were associated with treatment efficacy for depressive severity, suicidal ideation, and anxiolytic response to intravenous ketamine. Missing data were imputed via the last observation carried forward. The model controlled for sex and age.

## Results

### Clinical characteristics

In total, 85 patients with treatment-resistant major depression who had previously received intravenous ketamine at the CRTCE were enrolled in this study. In all, 84 patients completed the genetics kit and were included in subsequent analyses. Patient demographics are reported in Table 1.

**Table 1.** Baseline demographics (N = 84).

Sex *n* (%)	
Female	55 (65.5)
Male	29 (34.5)
Age (SD)	43.11 (13.34)
Race *n* (%)	
Caucasian	74 (88.1)
South Asian	5 (5.9)
Latin American	3 (3.6)
Middle Eastern	1 (1.2)
Native American	1 (1.2)
Highest level of education *n* (%)	
<High school	2 (2.4)
High school graduate	9 (10.7)
Some University/College	27 (32.1)
Associate’s degree	3 (3.6)
Bachelor’s degree	23 (27.4)
Graduate degree	9 (10.7)
Professional degree	11 (13.1)
Marital status *n* (%)	
Single	37 (44.0)
Married	31 (36.9)
Separated	6 (7.1)
Common law	5 (6.0)
Divorced	4 (4.8)
Widowed	1 (1.2)
Val66Met status *n*(%)	
Val/Val	54 (64.3)
Val/Met	29 (34.5)
Met/Met	1 (1.2)
CYP2B6 metabolizer status *n*(%)	
Poor metabolizer	5 (6.0)
Intermediate metabolizer	34 (40.5)
Normal metabolizer	41 (48.8)
Rapid metabolizer	4 (4.7)
Mean QIDS-SR16 (SD)	
Baseline score	17.4 (4.4)
Post infusion 4	11.7 (5.0)
Mean GAD-7 (SD)	
Baseline score	12.6 (5.5)
Post infusion 4	8.7 (5.6)

### Treatment effects

**Figure 1.**
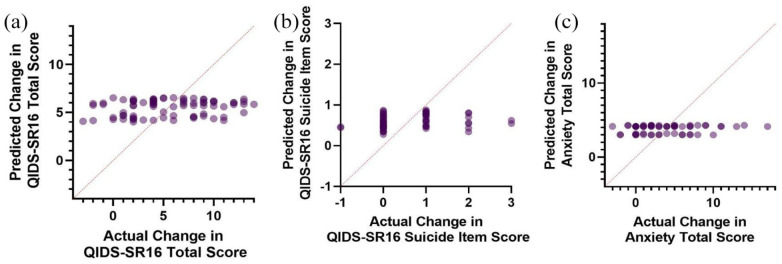
Predicted Val66Met regression model scores versus actual scores of (a) change in depressive severity, (b) change in suicidal ideation, and (c) change in anxiety severity.

There was a main effect of treatment on QIDS-SR16 total score (F(4, 75.5) = 37.7, *p* < 0.001). Depressive symptoms of participants significantly improved from baseline to post-infusions 2, 3, 4, and follow-up (*p*s < 0.001). Similarly, there was a main effect of treatment on suicidal ideation (*F*(4, 76.4) = 12.9, *p* < 0.001), with participants significantly reporting a reduction in their symptoms from baseline to all subsequent time points (*p*s < 0.01). Finally, there was a main effect of treatment on anxiety (*F*(2, 67.1) = 33.3, *p* < 0.001). Bonferroni’s corrected results indicated that anxiety improved from baseline to post-infusion four and follow-up visits (*p*s < 0.001). Figure 1 presents the corrected change in symptomatology over time. Overall, 22 of 85 participants met response criteria (i.e., reduction of QIDS-SR16 Total Score ⩾ 50%) and 13 participants met remission criteria (i.e., QIDS-SR16 Total Score ⩽ 5).

### Regression models of Val66Met and CYP2B6

**Figure 2.**
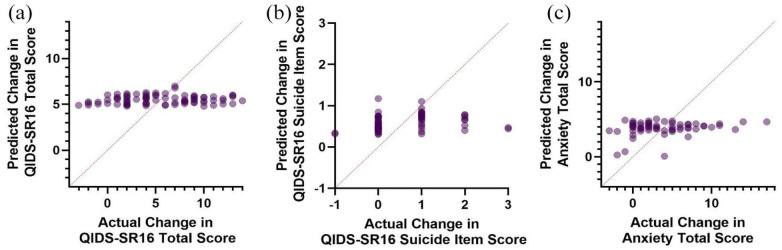
Predicted BDNF regression model scores versus actual scores of (a) change in depressive severity, (b) change in suicidal ideation, and (c) change in anxiety severity.

Multiple regression was conducted to predict the change in QIDS-SR16 from baseline to endpoint from the Val66Met genotype, sex, and age. Linearity was assessed by partial regression plots and there was no evidence of multicollinearity among the independent variables (variance inflation factor < 1.04). Normality was assessed by visual inspection of a P-P plot of standardized residuals. The multiple regression indicated that the Val66Met genotype, age, and sex were unable to significantly predict the change in QIDS-SR16 total score *F*(3, 76) = 0.97, *p* = 0.41 (Figure 1(a)). A second multiple regression was conducted predicting change in QIDS-SR16 by the CYP2B6 metabolizer status, sex, and age (Figure 2(a)). Assumptions of the test were assessed as previously described. Similarly, these predictors were unable to significantly predict changes in depression severity (*F*(5, 74) = 0.18, *p* = .97). Suicidality was calculated from the baseline to endpoint change in the SI item of the QIDS-SR16 (Figures 1(b) and 2(b)). Similarly, neither the models containing Val66Met (*F*(3, 59) = 0.73, *p* = 0.53) nor CYP2B6 (*F*(5, 57) = 0.61, *p* = 0.70) successfully predicted improvements in suicidality. When examining the anxiolytic properties of ketamine, Val66Met (*F*(3, 58) = 0.34, *p* = 0.80) and CYP2B6 (*F*(5, 56) = 0.63, *p* = 0.68) did not significantly predict response (Figures 1(c) and 2C). Finally, the average dissociation score was calculated by averaging the CADSS score across the four infusions. Val66Met (*F*(3, 61) = 0.66, *p* = .58) and CYP2B6 (*F*(5, 59) = 0.87, *p* = 0.51) status was unable to predict dissociation experienced by patients.

## Discussion

Overall, the foregoing study was unable to provide evidence that the Val66Met or CYP2B6 genotypes significantly predict antidepressant, anti-suicidal, anxiolytic, or average dissociative response in patients with treatment-resistant major depression receiving repeated intravenous ketamine. Although some evidence has suggested that CYP2B6 metabolizer status and BDNF allele expression may differentially affect the antidepressant response from a single infusion, to the author’s knowledge, this is the first study to report that these genotypes are not as relevant for individuals receiving repeated infusions (Chen et al., 2019; Laje et al., 2012). The findings from this study suggest that pharmacogenetic testing may not provide clinicians with predictive information for individuals receiving repeated infusions of intravenous ketamine.

This study’s finding that the Val66Met polymorphism in the BDNF gene was unrelated to improvements in patient symptomatology comports with findings from Su et al. (2017). While it has been hypothesized that the lower functioning Met allele may attenuate ketamine’s antidepressant function, to date, all studies that evaluated this relationship have been single-dose infusions at a strength ranging between 0.2 and 0.5 mg/kg (Chen et al., 2019; Laje et al., 2012). Clinical practice guidelines have suggested that repeated ketamine infusions ranging from 0.5 to 1.0 mg/kg convey superior clinical outcomes compared to lower dosing regimens (McIntyre et al., 2021a). As the foregoing study utilized a repeated dosing protocol at a higher strength than previous studies, it remains a testable hypothesis whether or not more frequent exposure to ketamine can offset deficiencies of the Met polymorphism.

This study also reports that the metabolizer status of CYP2B6 was unable to meaningfully predict improvements in depressive and anxiety symptoms as well as dissociation. These findings comport with Zarate et al. who found that there was no significant impact of the CYP450 family, including CYP2B6 (Zarate et al., 2012). While some studies have reported a significant association between CYP2B6 polymorphisms and ketamine antidepressive response, our population presented with significant polypharmacy, which was taken adjunctively with ketamine. Given the large number of medications metabolized by CYP2B6, other medications may interfere with the metabolism of the drug. It should be noted however that all patients were required to pause their medication regimens 6 h before their infusions and could restart post-infusion. Moreover, examination of the hepatic glucuronidation pathway may be warranted given approximately 80% of ketamine is metabolized and excreted as glucuronic-acid labile conjugates (Dinis-Oliveira, 2017). It should be further noted that the route of administration of ketamine in this study was through intravenous injection, resulting in a 99% bioavailability. It is possible that the CYP450 enzymes and the hepatic glucuronidation pathway that metabolize ketamine are not as effective due to the absence of the first-pass effect (Clements et al., 1982). Comparatively, the foregoing study cannot be generalized to patients receiving oral ketamine, as the bioavailability is approximately 16% and ketamine is rapidly metabolized into norketamine and dehydronorketamine (Clements et al., 1982; Dinis-Oliveira, 2017).

It should be noted that patients enrolled in the foregoing pharmacogenetics study exhibited a commensurate improvement in depression, suicidality, and anxiety symptoms compared to the full sample of patients receiving treatment at the CRTCE (McIntyre et al., 2020b). Overall 25% of the sample were responders to the treatment, with 15% achieving remission. This is notable given that the foregoing sample represented patients with a highly intractable form of TRD. The average number of unsuccessful past medication trials in this sample was approximately seven.

A significant strength of this study was that it was conducted completely remotely due to restrictions from the COVID-19 pandemic. All 85 participants who were enrolled in the study were able to complete the genetics kits and the questionnaires from their homes. Given the strict restrictions to non-essential in-person research visits, this study demonstrates the feasibility of conducting pharmacogenetics research remotely. Over 96% of participants in this study reported that the genetics kit was user-friendly and easy to use. An additional strength of this study includes the high representativeness of patients with treatment-resistant major depression, as there are minimal exclusion criteria for patients enrolled to receive ketamine compared to randomized control trials. Furthermore, the foregoing study utilized the QIDS-SR_16_ and GAD-7 self-report measures. Validation studies have indicated that these measures have strong conceptual coverage of depression and anxiety symptomatology (Rush et al., 2003; Spitzer et al., 2006). Importantly, these scales are sensitive to change from monoaminergic treatments (Johnson et al., 2019; Rush et al., 2005). Given the negative finding reported herein, it remains a testable hypothesis whether scales that measure rapid-onset treatments, such as the McIntyre and Rosenblat Rapid Response Scale, would be more sensitive to genetic markers (McIntyre et al., 2021b).

A significant limitation of this study is that it is retrospective, without a control group comparator. While ketamine has been well tolerated by the majority of patients who receive treatment at the CRTCE, this study only recruited patients who completed a set of four infusions and therefore is unable to evaluate patients who drop out due to intolerability (Rodrigues et al., 2020). Another limitation of this study is that blood biomarkers were not taken post-treatment and therefore plasma concentrations of BDNF and ketamine metabolites can only be proxied through the participant’s genotype. It should be further noted that dissociation was measured with the CADSS, which was initially developed to capture the phenomenology of dissociative symptoms in patients with post-traumatic stress disorder. Therefore, it may not adequately capture ketamine-induced dissociation as a function of CYP2B6 function. Another limitation is the generalizability of this study to other formulations of ketamine. It can be hypothesized that pharmacogenetics may provide insights into oral and intranasal formulations that may be more susceptible to first-pass mechanisms, as the bioavailability is less compared to intravenous injection. Finally, as data for this trial were collected from a real-world population receiving intravenous ketamine, there was missing data for 18 individuals who were imputed using LOCF.

Taken together, these results provide evidence that patients with the different genotypes from the Val66Met and the CYP2B6 genes did not significantly impact the antidepressant, anti-suicidal, and anxiolytic response of intravenous ketamine. Future studies should look to examine the effects of repeated infusions of ketamine and its metabolites at varying strengths. For example, the inclusions of blood markers that examine ketamine metabolites (i.e., norketamine and dehydronorketamine) at several post-infusion time points may provide additional information on whether CYP2B6 status affects ketamine metabolism. It remains testable whether individuals who metabolize ketamine slower may require a lower dose of intravenous ketamine to achieve the same antidepressant effect.

## References

[bibr1-02698811241238284] Autry AE Adachi M Nosyreva E , et al. (2011) NMDA receptor blockade at rest triggers rapid behavioural antidepressant responses. Nature 475: 91–95. 21677641 10.1038/nature10130 PMC3172695

[bibr2-02698811241238284] Berardelli I Serafini G Cortese N , et al. (2020) The involvement of hypothalamus-Pituitary-Adrenal (HPA) axis in suicide risk. Brain Sci 10: 653. 32967089 10.3390/brainsci10090653 PMC7565104

[bibr3-02698811241238284] Chen M-H Lin W-C Wu H-J , et al. (2019) Antisuicidal effect, BDNF Val66Met polymorphism, and low-dose ketamine infusion: Reanalysis of adjunctive ketamine study of Taiwanese patients with treatment-resistant depression (AKSTP-TRD). J Affect Disord 251: 162–169. 30925267 10.1016/j.jad.2019.03.075

[bibr4-02698811241238284] Clements JA Nimmo WS Grant IS (1982) Bioavailability, pharmacokinetics, and analgesic activity of ketamine in humans. J Pharm Sci 71: 539–542. 7097501 10.1002/jps.2600710516

[bibr5-02698811241238284] Coyle CM Laws KR (2015) The use of ketamine as an antidepressant: A systematic review and meta-analysis. Hum Psychopharmacol 30: 152–163. 25847818 10.1002/hup.2475

[bibr6-02698811241238284] Desta Z Gammal RS Gong L , et al. (2019) Clinical pharmacogenetics implementation consortium (CPIC) guideline for CYP2B6 and efavirenz-containing antiretroviral therapy. Clin Pharm Therap 106: 726–733. 10.1002/cpt.1477 PMC6739160 31006110

[bibr7-02698811241238284] Di Vincenzo JD Lipsitz O Rodrigues NB , et al. (2022) Frequency analysis of symptomatic worsening following ketamine infusions for treatment resistant depression in a real-world sample: Results from the canadian rapid treatment center of excellence. Psychiatr Res 307: 114321. 10.1016/j.psychres.2021.114321 34890909

[bibr8-02698811241238284] Dinis-Oliveira RJ (2017) Metabolism and metabolomics of ketamine: A toxicological approach. Forensic Sci Res 2: 2–10. 30483613 10.1080/20961790.2017.1285219 PMC6197107

[bibr9-02698811241238284] Dowlati Y Herrmann N Swardfager W , et al. (2010) A meta-analysis of cytokines in major depression. Biol Psychiatr 67: 446–457. 10.1016/j.biopsych.2009.09.033 20015486

[bibr10-02698811241238284] Duman RS Sanacora G Krystal JH (2019) Altered connectivity in depression: GABA and glutamate neurotransmitter deficits and reversal by novel treatments. Neuron 102: 75–90. 30946828 10.1016/j.neuron.2019.03.013 PMC6450409

[bibr11-02698811241238284] Dutton M Can AT Lagopoulos J , et al. (2022) Stress, mental disorder and ketamine as a novel, rapid acting treatment. Eur Neuropsychopharm 65: 15–29. 10.1016/j.euroneuro.2022.09.006 36206584

[bibr12-02698811241238284] Ellis JA Ong B (2017) The MassARRAY® system for targeted SNP genotyping. Meth Mol Biol 1492: 77–94. 10.1007/978-1-4939-6442-0_5 27822857

[bibr13-02698811241238284] Fukumoto K Fogaça MV Liu R-J , et al. (2019) Activity-dependent brain-derived neurotrophic factor signaling is required for the antidepressant actions of (2R,6R)-hydroxynorketamine. Proc Natl Acad Sci USA 116: 297–302. 30559184 10.1073/pnas.1814709116 PMC6320534

[bibr14-02698811241238284] Guze SB Robins E (1970) Suicide and primary affective disorders. Brit J Psychiatr J Ment Sci 117: 437–438. 10.1192/bjp.117.539.437 5481206

[bibr15-02698811241238284] Hijazi Y Boulieu R (2002) Contribution of CYP3A4, CYP2B6, and CYP2C9 isoforms to N-demethylation of ketamine in human liver microsomes. Drug Metabol Dispos 30: 853–858. 10.1124/dmd.30.7.853 12065445

[bibr16-02698811241238284] Johnson SU Ulvenes PG Øktedalen T , et al. (2019) Psychometric properties of the general anxiety disorder 7-item (GAD-7) scale in a heterogeneous psychiatric sample. Front Psychol 10: 1713. 31447721 10.3389/fpsyg.2019.01713 PMC6691128

[bibr17-02698811241238284] Laje G Lally N Mathews D , et al. (2012) Brain-derived neurotrophic factor Val66Met polymorphism and antidepressant efficacy of ketamine in depressed patients. Biol Psychiatr 72: e27–e28. 10.1016/j.biopsych.2012.05.031 PMC3786174 22771240

[bibr18-02698811241238284] Lüscher C Malenka RC (2012) NMDA receptor-dependent long-term potentiation and long-term depression (LTP/LTD). Cold Spring Harbor Perspect Biol 4: a005710. 10.1101/cshperspect.a005710 PMC3367554 22510460

[bibr19-02698811241238284] McEwen BS Stellar E (1993) Stress and the individual. Mechanisms leading to disease. Arch Intern Med 153: 2093–2101. 8379800

[bibr20-02698811241238284] McIntyre RS Carvalho IP Lui LMW , et al. (2020) The effect of intravenous, intranasal, and oral ketamine in mood disorders: A meta-analysis. J Affect Disord 276: 576–584. 32871689 10.1016/j.jad.2020.06.050

[bibr21-02698811241238284] McIntyre RS Rodrigues NB Lee Y , et al. (2020) The effectiveness of repeated intravenous ketamine on depressive symptoms, suicidal ideation and functional disability in adults with major depressive disorder and bipolar disorder: Results from the Canadian rapid treatment center of excellence. J Affect Disord 274: 903–910. 32664031 10.1016/j.jad.2020.05.088

[bibr22-02698811241238284] McIntyre RS Rosenblat JD Nemeroff CB , et al. (2021) Synthesizing the evidence for ketamine and esketamine in treatment-resistant depression: An international expert opinion on the available evidence and implementation. Am J Psychiatry 178: 383–399. 33726522 10.1176/appi.ajp.2020.20081251 PMC9635017

[bibr23-02698811241238284] McIntyre RS Rodrigues NB Lipsitz O , et al. (2021) Validation of the McIntyre and rosenblat rapid response scale (MARRRS) in adults with treatment-resistant depression receiving intravenous ketamine treatment. J Affect Disord 288: 210–216. 33965843 10.1016/j.jad.2021.03.053

[bibr24-02698811241238284] Meshkat S Rodrigues NB Di Vincenzo JD , et al. (2021) Pharmacogenomics of ketamine: A systematic review. J Psychiatr Res 145: 27–34. 34844049 10.1016/j.jpsychires.2021.11.036

[bibr25-02698811241238284] Rodrigues NB McIntyre RS Lipsitz O , et al. (2020) Safety and tolerability of IV ketamine in adults with major depressive or bipolar disorder: Results from the Canadian rapid treatment center of excellence. Exp Opin Drug Safety 19: 1031–1040. 10.1080/14740338.2020.1776699 32539491

[bibr26-02698811241238284] Rong C Park C Rosenblat JD , et al. (2018) Predictors of response to ketamine in treatment resistant major depressive disorder and bipolar disorder. Int J Environ Res Public Health 15: 771. 29673146 10.3390/ijerph15040771 PMC5923813

[bibr27-02698811241238284] Rush AJ Trivedi MH Ibrahim HM , et al. (2003) The 16-item quick inventory of depressive symptomatology (QIDS), clinician rating (QIDS-C), and self-report (QIDS-SR): A psychometric evaluation in patients with chronic major depression. Biol Psychiatr 54: 573–583. 10.1016/s0006-3223(02)01866-8 12946886

[bibr28-02698811241238284] Rush AJ Trivedi MH Carmody TJ , et al. (2005) Self-reported depressive symptom measures: Sensitivity to detecting change in a randomized, controlled trial of chronically depressed, nonpsychotic outpatients. Neuropsychopharmacology 30: 405–416. 15578008 10.1038/sj.npp.1300614

[bibr29-02698811241238284] Rush AJ Trivedi MH Wisniewski SR , et al. (2006) Acute and longer-term outcomes in depressed outpatients requiring one or several treatment steps: A STAR* D report. Am J Psychiatr 163: 1905–1917. 17074942 10.1176/ajp.2006.163.11.1905

[bibr30-02698811241238284] Singh JB Fedgchin M Daly E , et al. (2016) Intravenous esketamine in adult treatment-resistant depression: A double-blind, double-randomization, placebo-controlled study. Biol Psychiatr 80: 424–431. 10.1016/j.biopsych.2015.10.018 26707087

[bibr31-02698811241238284] Slavich GM Irwin MR (2014) From stress to inflammation and major depressive disorder: A social signal transduction theory of depression. Psychol Bull 140: 774–815. 24417575 10.1037/a0035302 PMC4006295

[bibr32-02698811241238284] Spitzer RL Kroenke K Williams JBW , et al. (2006) A brief measure for assessing generalized anxiety disorder: The GAD-7. Arch Intern Med 166: 1092–1097. 16717171 10.1001/archinte.166.10.1092

[bibr33-02698811241238284] Su T-P Chen M-H Li C-T , et al. (2017) Dose-related effects of adjunctive ketamine in taiwanese patients with treatment-resistant depression. Neuropsychopharmacology 42: 2482–2492. 28492279 10.1038/npp.2017.94 PMC5686503

[bibr34-02698811241238284] Trivedi MH Rush AJ Wisniewski SR , et al. (2006) Evaluation of outcomes with citalopram for depression using measurement-based care in STAR* D: Implications for clinical practice. Am J Psychiatr 163: 28–40. 16390886 10.1176/appi.ajp.163.1.28

[bibr35-02698811241238284] Wang W Liu L Yang X , et al. (2019) Ketamine improved depressive-like behaviors via hippocampal glucocorticoid receptor in chronic stress induced- susceptible mice. Behav Brain Res 364: 75–84. 30753876 10.1016/j.bbr.2019.01.057

[bibr36-02698811241238284] Yanagihara Y Kariya S Ohtani M , et al. (2001) Involvement of CYP2B6 in n-demethylation of ketamine in human liver microsomes. Drug Metabol Dispos 29: 887–890. 11353758

[bibr37-02698811241238284] Zarate CA Jr Brutsche N Laje G , et al. (2012) Relationship of ketamine’s plasma metabolites with response, diagnosis, and side effects in major depression. Biol Psychiatr 72: 331–338. 10.1016/j.biopsych.2012.03.004 PMC3442255 22516044

